# Ruminal methane emissions, metabolic, and microbial profile of Holstein steers fed forage and concentrate, separately or as a total mixed ration

**DOI:** 10.1371/journal.pone.0202446

**Published:** 2018-08-15

**Authors:** Rajaraman Bharanidharan, Selvaraj Arokiyaraj, Eun Bae Kim, Chang Hyun Lee, Yang Won Woo, Youngjun Na, Danil Kim, Kyoung Hoon Kim

**Affiliations:** 1 Department of International Agricultural Technology, Graduate School of International Agricultural Technology, Seoul National University, Pyeongchang, Gangwon, The Republic of Korea; 2 Department of Ecofriendly Livestock Science, Institute of Green Bio Science and Technology, Seoul National University, Pyeongchang, Gangwon, The Republic of Korea; 3 Department of Animal Life Science, Kangwon National University, Chuncheon, The Republic of Korea; 4 Department of Animal Science and Technology, Konkuk University, Seoul, The Republic of Korea; 5 Department of Farm Animal Medicine, College of Veterinary Medicine, Seoul National University, Seoul, The Republic of Korea; The University of Sydney, AUSTRALIA

## Abstract

Few studies have examined the effects of feeding total mixed ration (TMR) versus roughage and concentrate separately (SF) on ruminant methane production. Therefore, this study compared differences in methane production, ruminal characteristics, total tract digestibility of nutrients, and rumen microbiome between the two feeding methods in Holstein steers. A total six Holstein steers of initial bodyweights 540 ± 34 kg were divided into two groups and assigned to a same experimental diet with two different feeding systems (TMR or SF) in a crossover design with 21 d periods. The experimental diet contained 73% concentrate and 27% forage and were fed twice a day. The total tract digestibility of crude protein, neutral detergent fibre, and organic matter were not affected by the two different feeding systems. Steers fed TMR emitted more methane (138.5 vs. 118.2 L/d; P < 0.05) and lost more gross energy as methane energy (4.0 vs. 3.5% gross energy intake; P = 0.005) compared to those fed SF. Steers fed TMR had greater (P < 0.05) total volatile fatty acid (VFA), ammonia-N concentrations and propionate proportion of total VFA at 1.5 h, whereas lower after that compared to steers fed SF. The greater (P < 0.05) acetate: propionate ratio at 4.5 h for steers fed TMR reflected the shift of H_2_ sink from propionate towards acetate synthesis. The lower (P < 0.05) isobutyrate and isovalerate proportions of total VFA observed in steers fed TMR implies decrease in net consumption of H_2_ for microbial protein synthesis compared to SF. There were no differences in both major bacterial and archaeal diversity between TMR and SF, unlike several minor bacterial abundances. The minor groups such as *Coprococcus*, *Succiniclasticum*, *Butyrivibrio*, and *Succinivibrio* were associated with the changes in ruminal VFA profiles or methanogenesis indirectly. Overall, these results indicate that SF reduces methane emissions from ruminants and increases propionate proportion of total VFA without affecting total tract digestion compared to TMR. There were no evidences that the response differed due to different major underlying microbial population.

## Introduction

Greenhouse gas emissions from livestock production are expected to increase over the coming decades due to the projected increase in demand for livestock products [1]. Besides its negative impact on the environment, the methanogenesi**s** process represents a loss of 3–10% of the gross energy intake of the animal and leads to the unproductive use of dietary energy [2]. Advances in understanding ruminant nutrition and rumen relevant to methane formation have led to various strategies to reduce CH_4_ emissions from the rumen; the use of chemical inhibitors such as bromochloromethane; electron receptors such as fumarate, nitrates, sulphates, and nitro ethane; and bioactive plant compounds such as tannin and saponin [3] [4]. However, the use of these compounds as feed additives has not been promising because of several adverse effects, such as a reduction in fibre digestibility and feed intake, toxicity to the rumen microbiome, and questions regarding the persistence of the effect.

Increasing the productivity of cattle to reduce CH_4_ emissions is a key area of interest because reducing the ruminant population being farmed is not an option. Hence, alternative feeding strategies such as total mixed ration (TMR) is of interest [3], because it has been reported to be of significant benefit in terms of increasing feed intake and digestibility; minimising choice feeding among individual feeds; and maintaining sufficient fibre intake to support rumen health, such as a stable ruminal pH and a lower A/P ratio [5] [6] compared to animals fed roughage and concentrates separately (SF). However, there were also contradictory reports that feeding a TMR had no effect on animal performance or the carcass traits of steers [7] and milk production and milk composition [8] [9].

Despite the importance of feeding system on livestock production and environmental impact, very few studies have compared the effects of SF and TMR feeding on CH_**4**_ production in ruminants and showed inconsistent results. Holter et al. [10] reported that yield of milk per unit total diet DM was about 4% more for TMR than for the same feeds offered separately but the CH_**4**_ yield (% GEI) was not affected significantly by treatments. However, Lee et al. [11] showed a significantly higher CH_**4**_ yield (% GEI) for TMR than for the same feeds offered separately. Hence, the effect of these feeding system on enteric CH_**4**_ production should be validated, since the TMR feeding practice is rising up both in developed and developing countries. So, the objective of this study was to confirm the advantages of TMR in terms of a stable ruminal pH and nutrients digestibility in Holstein steers fed same amount with the same ingredients, and to understand how its ruminal fermentation characteristics affected ruminal methane production and ruminal microbial communities using Next Generation sequencing technique.

## Materials and methods

All experimental animals were obtained from the Seoul National University animal farm where this experiment was performed. All experiments involving animals, methods and protocols were approved by the Committee for the Institutional Animal Care and Use of Seoul National University (SNU-160105-1), and all methods and protocols were carried out in accordance with the relevant guidelines and regulations. Animal feeds were purchased from a local feed mill company, and all experimental chemicals were obtained from Sigma*-*Aldrich (St. Louis, MO, USA)

### Animals and experimental design

Six Holstein steers of initial body weights 540 ± 34 kg were divided into two groups of three steers and assigned to two experimental diets (TMR or SF) in a crossover design with 21 d periods. Each period was comprised of 12 d for diet adaptation in the pen, 4 d for metabolic adaptation in the indirect respiratory chamber, 2 d for CH_4_ measurement, 2 d for fecal sampling, and 1 d for rumen fluid sampling.

### Experimental diet and feeding

Total mixed ration was prepared using 73% concentrates (including water, yeast culture, limestone, salt, and molasses) and 27% roughage on DM basis (Table 1). The moisture contents of the TMR and concentrates used for SF were 10% and 17%, respectively. The amount fed to the animals was restricted and adjusted to achieve average daily gains of 0.65 kg [12], and the animals were fed equal amounts twice a day at 0900 and 1800 h (Table A in S1 Table). In the SF system, the roughage was given first, and then the concentrate was given 40 mins later to prevent an unnecessary drop in pH during the initial ruminal fermentation. The animals were given full access to water and a mineral block *ad libitum* in the pen and respiratory chamber. Samples of the feed offered were collected and stored to measure the dry matter content and perform other chemical analyses.

**Table 1:** Ingredients and chemical composition of the basal diet used in the experiment.

Ingredient composition (g/kg fed basis)		Chemical composition ^1^	
Concentrate		DM (g/kg fed basis)	900
Corn gluten feed	230	OM	792
Wheat bran	10	CP	166
Coconut meal	135	EE	44
Broken corn	56	aNDFom^3^	287
Steam flaked corn	24	ADFom^4^	126
Cotton hull pellet	118	GE, MJ/kg DM	16.3
Water	20		
Whole cotton seed	40		
Yeast culture	30		
Limestone	10		
Salt	2		
Molasses	50		
Mineral-vitamin mixture^2^	5		
Roughage			
Alfalfa hay	50		
Perennial rye grass	100		
Annual rye grass	100		
Klein grass	2		

OM, Organic matter; CP, Crude protein; EE, Ether Extract; GE, Gross energy

^1^ Unless indicated otherwise, units are expressed as g/kg of DM.

^2^ The mineral-vitamin mixture includes vitamin A: 2,650,000 IU; vitamin D3: 530,000 IU; vitamin E: 1,050 IU; nicotinic acid: 10,000 mg; Fe: 13,200 mg; Mn: 4,400 mg; Zn: 4,400 mg; Copper: 2,200 mg; Iodine: 440 mg; Cobalt: 440 mg

^3^ Acid detergent fibre expressed excluding residual ash

^4^ Neutral detergent fibre assayed with a heat stable amylase and expressed exclusive of residual ash

### Measurement of methane emission

Methane production was measured using three indirect open-circuit whole-body respiratory chambers that had steel frames and were made of polycarbonate sheets [12]. Each chamber (137 × 256 × 200 cm wide × deep × tall) was equipped with a feeder, water bowl, air conditioner (model ALFFIZ-WBCAI-015H; Busung, India), dehumidifier (model DK-C-150E; Dryer, Korea) and circulatory fans to maintain the temperature and humidity. The gas analysis system consisted of a gas sampling pump (B.S. Technolab, Korea), a flow meter (model LS-3D; Teledyne Technologies, USA) consisted of a sealed rotary pump that provided a constant wet ventilation rate (wet VR) of 600 L/min, and a data acquisition and analysis unit equipped with a CH_4_ gas analyser (Airwell+7; KINSCO Technology, Korea) containing a tuneable diode LASER CH_4_ sensor with a range of 0 to 1000 ppm. The respiration chamber was maintained at a controlled temperature of 25°C and humidity of 50%. The gas analyser was calibrated, and the recovery rate of each chamber was tested at the beginning of the experiment using a standard CH_4_ gas mixture (25% mol/mol balance N_2_; Air Korea). Briefly, a fixed volume of CH_4_ (50 ml/min) was injected into each chamber from outside near the circulatory fan through a gas tube. The air was allowed to mix for 10 mins to achieve an equilibrium state with the inlet and outlet air passage closed. After that time, the inlet and outlet air passages were opened and the gas at both the inlet and outlet was sampled every 10.5 mins with the flush time and measuring time of 90 and 120 secs, respectively. The difference in CH_4_ concentration between inlet and outlet gas (DCH_4_ ppm), known dry standard temperate and pressure ventilation rate (Dry STP VR), and known injected CH_4_ concentration were used to calculate the gas recovery rates of the chamber using the formula

$$CH_{4}\;emission\;\left(L/min\right)=\left(Dry\;STP\;VR\times \left(\left[DCH_{4}\;ppm\right]/1000000\right)\right)/gas\;recovery\;rate$$

During the experiment, the same routine operation was carried out when the animals were placed in the chamber, and the CH_4_ emission was calculated using the same formula with known values. To avoid uncertainty in the data, we placed the same animals in the same chamber for both periods while measuring CH_4_ throughout the experiment. However, chamber limitation delayed the measurement of CH_4_ emission from the other group by 8 days.

### Digestion trial and rumen sampling

The effects of the feeding systems on the total tract digestibility of nutrients were studied using chromic oxide (Cr_2_O_3_) as an external marker. The feed was top-dressed with the marker twice daily at 0.2% of the daily feed amount for TMR, whereas it was mixed with the concentrate in the SF system before feeding. On day 19, a faecal grab sample (100 g fresh weight) was collected from the rectum of each animal 30 min before feeding and 1, 3, 5, and 7 h post feeding. On day 20, faeces were collected 30 min before and 2, 4, 6, and 8 h post feeding. The faeces samples were frozen at –20°C until analysed by steer within period. Samples of ruminal fluid were collected 1.5, 3, and 4.5 h post feeding on day 21 of each period using a stomach tube (Oriental dream cooperation, Korea). The ruminal fluids were squeezed through four layers of muslin and the pH was measured immediately using a pH meter (model AG 8603; Seven Easy pH, Mettler-Toledo, Schwerzenbach, Switzerland). For microbial analysis, the rumen fluid was stored at -80°C until DNA is extracted. The ruminal fluid was centrifuged at 11,200 ×g for 10 min (Centrifuge Smart 15, Hanil Science Industrial, South Korea), and the supernatant was transferred to a 50 mL centrifuge tube and stored at –20°C for determination of the ammonia-nitrogen (NH_3_-N) and volatile fatty acid (VFA) concentrations.

### Chemical analyses

The samples of feed and faeces were dried in a forced-air oven at 65°C for 72 h to estimate the dry matter (DM) content and ground to pass through a 1 mm screen (Thomas Scientific Model 4, New Jersey, USA); and then assayed for crude protein (CP) by combustion (Method 990.03; [13]) using an Elementar rapid N-cube protein/nitrogen apparatus (Elementar Americas, Mt. Laurel, NJ, USA), ash (Method 942.05, [13]), ether extract (EE; Method 960.39, [13]), and chromium (Method 990.09; [13]). The neutral detergent fibre content was assayed with a heat stable amylase and expressed exclusive of residual ash (aNDFom) using the method of Van Soest [14]. Contents of acid detergent fibre excluding residual ash (ADFom) was determined according to Van Soest [15]. The gross energy (GE) of both feed and faecal samples was estimated using a bomb calorimeter (Shimadzu CA-3, Shimadzu, Japan). A 5.0 mL aliquot of rumen fluid was mixed with 1.0 mL 25% HPO_3_ and 0.2 mL 2% pivalic acid to measure VFAs [16], and the mixture was analyzed using an Agilent 7890B GC system (Agilent Technologies, Santa Clara, CA, USA) with a FID detector. The inlet and detector temperature were maintained at 220 °C. Aliquots (1 μl) were injected with a split ratio of 10:1 into a 30 m × 0.25 mm × 0.25μm Nukol fused-silica capillary column (Cat. No: 24107, Supelco, Sigma*-*Aldrich, St. Louis, MO, USA) with helium carrier gas set to a flow rate of 1 mL/ min and initial oven temperature of 80 °C. The oven temperature was held constant at the initial temperature for 1 min, and thereafter increased at 20 °C /min to a temperature of 180 °C and held for 1 min, and increased at 10 °C /min to a final temperature of 200 °C, and a final run time of 14 min. The NH_3_-N concentration was determined using a modified colorimetric method [17]. The particle size of the feed in both the TMR and SF was determined using a Penn State particle separator using the technique of Kononoff et al. [18].

### DNA extraction, PCR and 16S rRNA gene sequencing

The 24 rumen samples collected at 3 time intervals from 4 Holstein steers fed by two different feeding system were thawed at room temperature and the genomic DNA was extracted from them using the NucleoSpin soil kit (Macherey-Nagel, Düren, Germany), with minor modifications. Briefly, 5 ml of thawed rumen fluid was centrifuged at 11,200 ×g using Centrifuge Smart 15 (Hanil Science Industrial, Seoul, South Korea) and supernatant was discarded. Three hundred and fifty μl of lysis buffer and 75 μl of enhancer was added to the pellet, and vortexed for 2 mins. The liquid was transferred to the NucleoSpin Bead Tube Type A containing the ceramic beads and was vortexed using the Taco Prep bead beater (GeneReach Biotechnology Corp., Taiwan). The rest of the procedure was followed according to the manufacturer’s instructions. Extracted DNA samples were stored at -20°C prior to PCR amplifications. In the present study, the forward primer F515 (5′-CACGGTCGKCGGCGCCATT-3′) and reverse primer R806 (5′-GGACTACHVGGGTWTCTAAT-3′) targeting the V4 domain of the bacterial/archaeal 16S rRNA was selected as target for interrogating the bacterial and archaeal communities since the genus-level coverage of this region was found to be high [19]. This primer set targets ~312 bp of the V4 hypervariable regions that can be fully covered by the Illumina MiSeq. The primer sets were modified to contain an Illumina adapter and linker region for sequencing on the Illumina MiSeq platform and, on the reverse primer, a 12-base barcode to enable sample multiplexing.

Briefly, the PCR reaction was prepared using genomic DNA (5 ng), reaction buffer with 25 mM Mg2+, dNTP (200 mM each), Ex Taq polymerase (0.75 units; Takara Bio, Shiga, Japan), and 5 pmol each of the barcoded primers. The PCR reaction was carried out at 94°C for 3 min for initial denaturation, 30 cycles of 45 s at 94°C, 1 min at 55°C, 90 s at 72°C for amplification, and 72°C for 10 min for final extension. Then, the PCR products were quantified using the Quant-iT^TM^ dsDNA High-Sensitivity Assay Kit (Invitrogen, CA, USA), and all amplicons from the 24 DNA samples were loaded onto a 1.5%-agarose gel. Bands were visualized and the target band was excised and extracted using QIAquick Gel Extraction Kit (Qiagen, Hilden, Germany). The extracted DNA was used to construct the V4 sequencing library with the NEBNext Ultra DNA Library Prep Kit (Cat. No: E7370S; New England Biolabs, Ipswich, MA, USA), according to the manufacturer’s instructions and the library was sequenced for paired-end 250-bp reads in the Illumina MiSeq.

### Bioinformatics and statistical analysis

The raw Illumina MiSeq reads were demultiplexed according to the barcodes and the sequences were quality-filtered (> = Q20). The processed paired reads were concatenated into a single read, and each single read was screened for operational taxonomic unit (OTU) picking using the UCLUST embedded within the QIIME 1.9.0 with the greengenes database (gg_otus-13_8-release, 97% nucleotide identity). Alpha diversity indices including Chao1, Shannon and Simpson indices were estimated using the PAST software [20], and the reads were rarified based on mean values of 10 iterations with 10,000 reads per sample. To identify bacterial lineages that drive the clustering of microbial communities in both feeding system, we performed PCA (Principal Component Analysis) using the fviz_pca_biplot function in the FactoMineR package of R [21]. Non-parametric Kendall rank correlation was used to test the correlation between the mean production variables and bacterial communities in rumen fluid using the corr.test function in the psych package of R [22]. The resulting correlation matrix was visualized in a heatmap format using the plot_ly function in the plotly package of R [23].

Data on daily methane emissions and total tract digestibility were analysed using the MIXED procedure of SAS (SAS Institute, Cary, NC, USA). The model included a fixed effect of dietary treatment and the random effects of period and animal nested within treatment. Ruminal fermentation characteristics and microbial diversity were analysed as repeated measures using SAS PROC MIXED [24]. The fixed effects in the model were dietary treatment and fermentation time as well as the interaction between them. Each animal within treatment were considered as a random effect. Appropriate covariance structures were chosen based on Akaike information criterion. Means were calculated using the LSMEANS statement, and the animal was considered the experimental unit. Treatment differences were considered significant at P < 0.05.

## Results

### Feed Intake, nutrient digestibility, ruminal methane production and fermentation

The steers fed TMR and SF had similar DM intakes (Table A in S1 Table) and nutrient digestibility (Table B in S1 Table). The steers fed TMR produced more (P < 0.05) CH_4_ (g/d, g/kg DMI, g/kg OMI and %GEI) than those fed SF (Table 2). As expected, the TMR mixing process increased the percentage of particles less than 1.18 mm and decreased the percentage of particles >19 mm (P < 0.05 and P = 0.01, respectively) (Table 3). The two different feeding systems had distinctly different ruminal fermentation characteristics. Ruminal pH, acetate, and the acetate:propionate ratio were lower for TMR feeding than for the SF system at 1.5 h but were greater (P < 0.05) for TMR at 4.5 h (Table 4). In contrast, the overall pattern for the other variables, total VFA, and propionate was greater (P < 0.05) in steers fed TMR than those fed SF at 1.5 h, whereas the inverse was observed 4.5 h post feeding. No difference (P > 0.05) in the proportion of butyrate in total VFA was noted between the feeding systems. The concentration of NH_3_-N (P = 0.005), and the proportions of isobutyrate and isovalerate in total VFA in SF were also greater (P < 0.01) than those in TMR after feeding (Table 4).

**Table 2:** Least square means of methane production recorded in steers fed roughage and concentrate either as total mixed ration (TMR) or separately (SF) over 24 h (*n* = 6).

Item	TMR	SF	SEM	*P*-value
CH_4_, L/day	138.5	118.2	3.2	0.029
CH_4_, g/day	96.1	84.4	2.3	0.029
CH_4_, g/kg DMI	11.3	10.3	0.1	0.011
CH_4_, g/kg OMI	13.4	11.6	0.5	0.054
CH_4_, g/kg DOM	20.7	20.1	0.5	0.338
CH_4_ E, % GEI	4.0	3.5	0.0	0.005

DMI, dry matter intake; OMI, organic matter intake; DOM, digestible organic matter; GEI, gross energy intake

**Table 3:** Feed particle size distribution (%) in the total mixed ration (TMR) and separate feed (SF).

Particle size (g/kg DM)	TMR	SF	SEM	*P*-value
>19 mm	54	181	23.7	0.012
19–8.0 mm	294	234	6.5	0.002
8.0–1.18 mm	355	357	11.2	0.890
<1.18 mm	297	229	16.3	0.026

**Table 4:** Effects of TMR and SF system on ruminal fermentation characteristics^1^.

Time interval^2^	1.5 h		3 h		4.5 h		SEM	P-value (FS)	P-value (Time)		
Item/Feed type	TMR	SF	TMR	SF	TMR	SF			1.5 h	3 h	4.5 h
Volatile fatty acids	** **	** **	** **	** **	** **	** **	** **	** **	** **	** **	** **
Total VFA (mM)	122.8	95.4	114.4	118.9	104.7	126.8	9.7	0.962	0.009	0.650	0.032
Acetate, %	55.0	58.4	56.0	54.0	58.6	55.3	1.2	0.337	0.007	0.103	0.008
Propionate, %	25.0	22.5	24.0	25.2	22.5	24.1	0.9	0.876	0.008	0.160	0.088
Butyrate, %	12.7	11.8	13.2	13.0	13.1	13.2	0.9	0.649	0.332	0.877	0.977
Isobutyrate, %	0.6	1.3	0.6	1.2	0.5	1.3	0.1	< .0001	< .0001	< .0001	< .0001
Valerate, %	1.9	1.4	2.0	1.8	1.7	1.9	0.1	0.048	< .0001	0.064	0.072
Isovalerate,%	4.7	4.5	4.1	4.6	3.4	4.2	0.2	0.027	0.352	0.110	0.006
Acetate: Propionate	2.2	2.6	2.3	2.2	2.6	2.3	0.1	0.921	0.001	0.124	0.038
NH_3_-N, mg/L	18.9	9.2	14.9	14.8	9.4	15.1	1.8	0.124	< .0001	0.925	0.005
pH	6.5	6.7	6.5	6.5	6.6	6.3	0.1	0.899	0.067	0.808	0.060

^1^ Values are LS means with standard error

^2^ Sampling time after morning feeding; TMR- Total Mixed Ration; SF- Separate Feeding; SEM- Standard Error of the Means

FS–Feeding system

### Richness, diversity estimates, and rumen bacteria and archaeal composition

Illumina sequencing produced a total of good quality 1,231,081 bacterial and 323,775 archaeal sequences from 24 samples from 4 Holstein steers. These sequences included an average of 51,295 bacterial reads ranging from 28,357 to 176,175 reads and 15,418 archaeal reads ranging from 6,910 to 27,395 reads per rumen sample. The feeding system was found to have no effect (P > 0.05) on the total reads generated in bacteria and archaea. The mean observed OTUs were 1,911 and 1,937 for SF and TMR, respectively at a depth of 10000 reads per sample. Alpha diversity metrics, Chao1, exhibited a difference (*P* < 0.05) denoting greater bacterial richness in SF system. However, Shannon and Simpson indices did not exhibit any significant differences in both bacterial and archaeal diversity between the feeding systems at any time interval (S2 Table).

**Fig 1:**
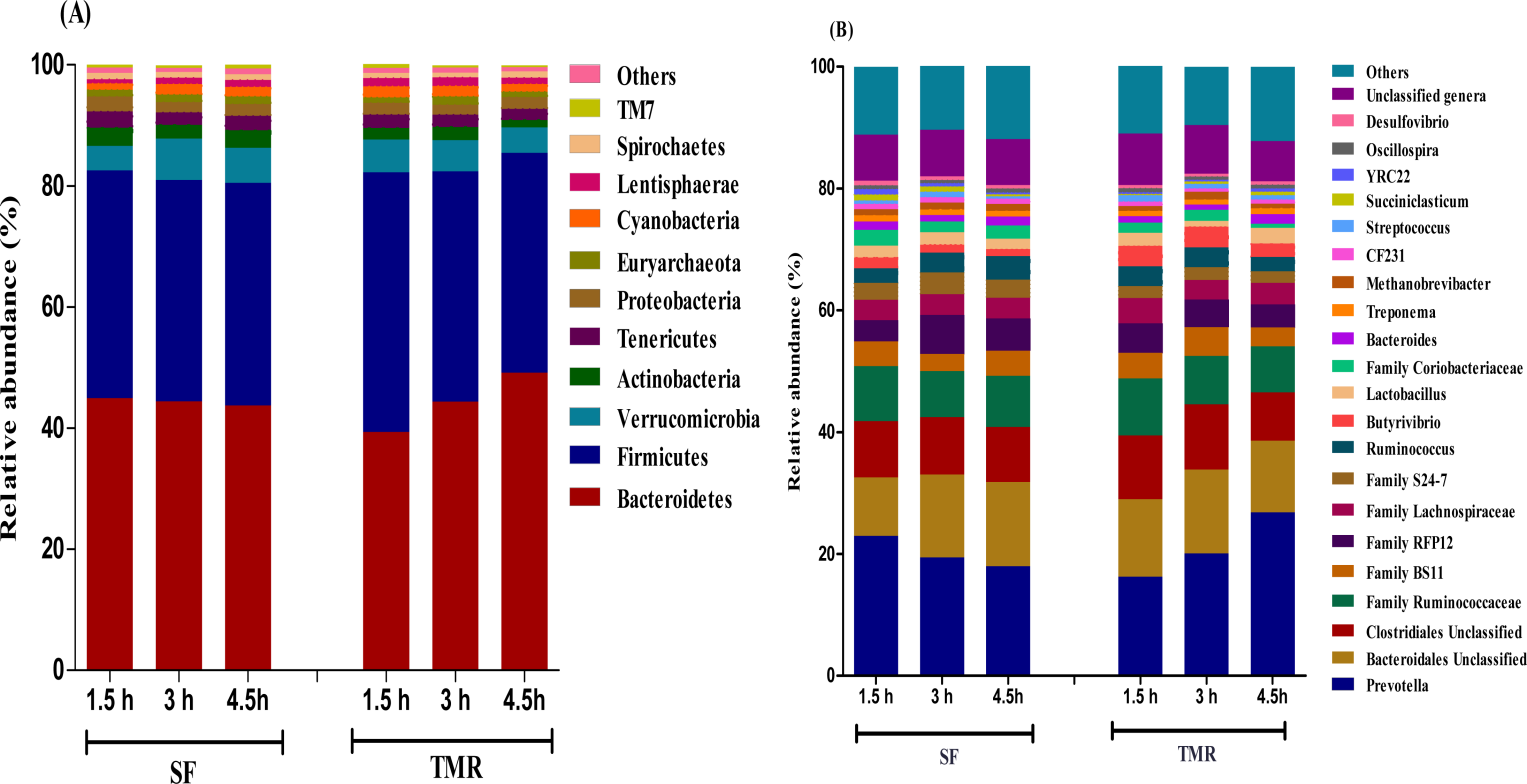
The taxonomic profiles for the relative phylum-level (A) and genus-level (B) abundance of bacteria and archaea in both feeding systems classified by representation at > 0.5% of total sequences.

The taxonomic analysis of the reads revealed the presence of 31 phyla, out of which 12 bacterial and 1 archaeal phylum had relative abundance > 0.1%. Phylum *Bacteroidetes* (40–50%) and *Firmicutes* (35–40%) were typically together representing around 80–85% of the total sequences in all samples (S3 Table; Fig 1A). Among other phyla, *Verrucomicrobia* (4–5%), *Actinobacteria* (1–3%), *Tenericutes* (1.8–2.6%), *Proteobacteria* (1.5–2.5%), *Cyanobacteria* (1–1.9%), *Lentisphaerae* (0.7–1.4%) and *Spirochaetes* (0.8–1%) were considerably more prevalent. *Fibrobacteres*, *Chloroflexi*, *Planctomycetes* and *TM7* were found to be in low abundance (0.1–1%). The archaeal phylum *Euryarchaeota* was observed to have 0.8–1.3% of relative abundance in both the feeding systems. At the genus level, 34 genera out of 374 genera detected had relative abundance > 0.1%. The more predominant population (relative abundance > 0.5%) in the *Bacteroidetes* phylum belonged to *Prevotella* (18–27%), *Bacteroides* (0.9–1.5%), *CF231* (0.6–0.9%) and *YRC22* (0.3–0.9%) (S3 Table; Fig 1B). A number of taxa were not classified to the genus level, but were present in great abundance. That includes Family *BS11* (2.7–4.7%), Family *S24-7* (1.9–3.5%) and order *Bacteroidales* (9.5–13.7%). Among the Firmicutes, *Ruminococcus* (2.4–3.9%), *Butyrivibrio* (1.15–3.38%), *Lactobacillus* (0.97–2.19%), *Oscillospira* (0.51–0.68%), *Streptococcus* (0.38–1.03%), *Succiniclasticum* (0.23–0.95%) and *Leuconostoc* (0.28–0.57%) were observed to be in high abundance (> 0.5%). In addition, *Desulfovibrio* (*Proteobacteria*) (0.54–0.75%) and *Treponema (Spirochaetes)* (0.8–1%) were also found to be predominantly high in both the feeding system. Likewise, only the genera *Methanobrevibacter* of the phylum *Euryarchaeota* had a relative abundance of (0.8–1.3%). Several abundances within the order of *Clostridiales* (7.95–10.45%), Family *Ruminococcaceae* (7.56–9.38%), *Lachnospiraceae* (3.17–4.16%) and *Christensenellaceae* (0.86–1.63%) were also prevalent.

### Differences in bacterial community composition between the feeding systems

**Fig 2:**
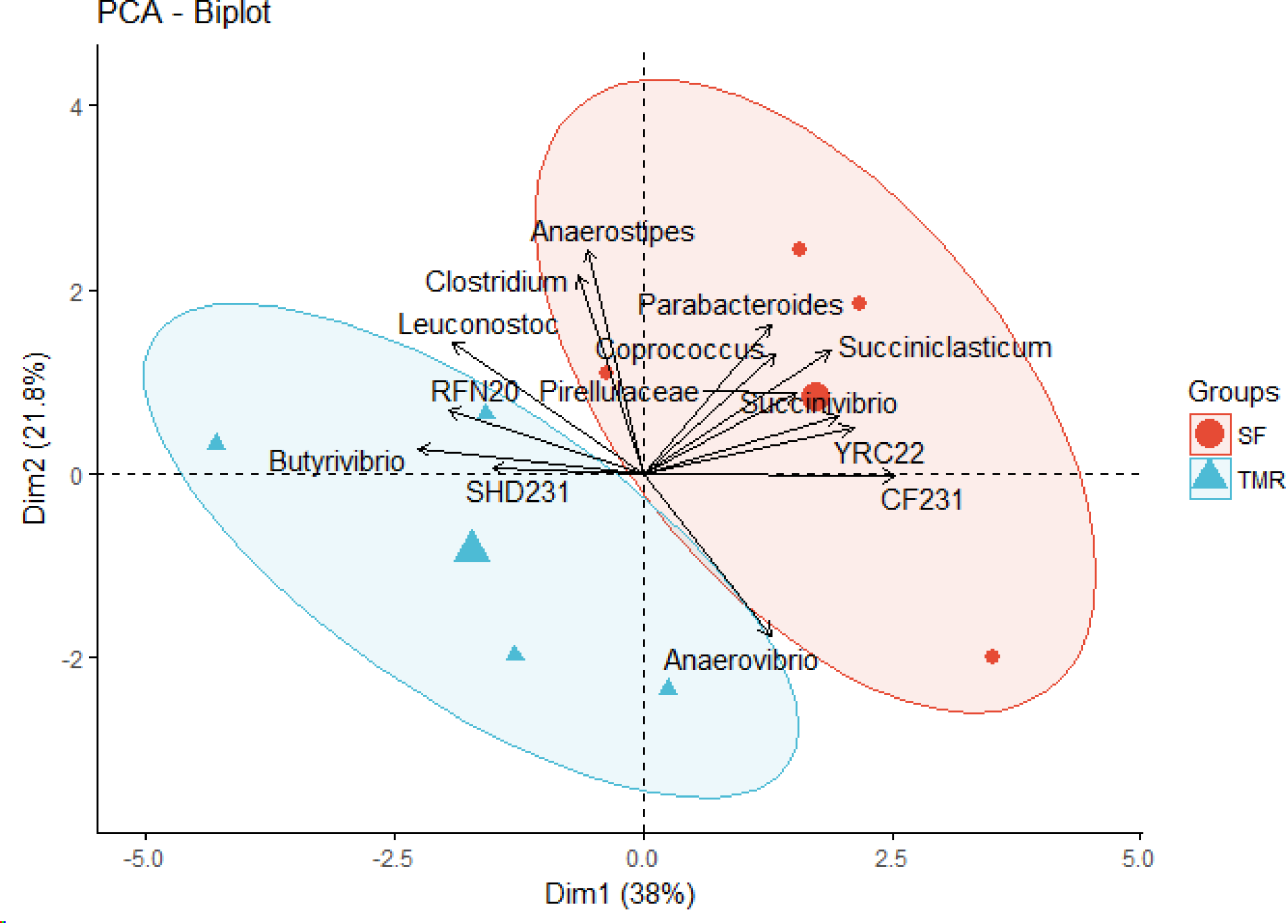
Principal component analysis (PCA) displaying correlations among the bacterial communities of the steers fed by two different feeding system.

The bacterial community composition between the feeding systems at the phylum level had no differences (P > 0.05). However, the abundance of the phylum *Actinobacteria* tended to vary (P = 0.061) in SF system (S3 Table). Likewise, at genera level, the mean abundance of *Parabacteroides* (P = 0.081), *YRC22* (P = 0.082), *Succiniclasticum* (P = 0.063), *Anaerovibrio* (P = 0.071) and *Succinivibrio* (P = 0.074) tended to be greater in SF system. Similarly, abundance of genera *CF231* and *Coprococcus* was greater (P < 0.05) in SF, whereas, abundance of *SHD-231* (P = 0.072), *Butyrivibrio* and *RFN20* was observed to be greater (P < 0.05) in TMR feeding system (Table 5; S3 Table). In the PCA plot, samples clustered in two groups by feeding system and were correlated to the mean taxonomic annotation. (Fig 2).

**Table 5:** Relative abundance of taxa in the steers fed by two feeding system representing > 0.1% of total sequences that tend to differ (P < 0.1) and significantly differ (P < 0.05).

Phylum	Classification	Percentage of total sequences ^1^^,^^2^						SEM	P value (FS)	P value (Time)		
		SF			TMR							
		1.5 h	3 h	4.5 h	1.5 h	3 h	4.5 h			1.5 h	3 h	4.5 h
**Bacteroidetes**	Parabacteroides	0.22	0.30	0.21	0.17	0.15	0.16	0.02	**0.081**	0.336	**0.026**	**0.084**
** **	Family Paraprevotellaceae; Genus CF231	0.88	0.88	0.82	0.71	0.58	0.67	0.06	**0.026**	0.549	0.214	0.266
** **	Family Paraprevotellaceae; Genus YRC22	0.90	0.50	0.29	0.26	0.29	0.55	0.07	**0.082**	0.155	0.112	0.184
**Firmicutes**	Butyrivibrio	1.74	1.29	1.15	3.31	3.38	2.21	0.67	**0.047**	0.409	0.179	0.425
** **	Succiniclasticum	0.95	0.84	0.34	0.23	0.41	0.54	0.11	**0.063**	0.132	0.514	0.139
	Coprococcus	0.28	0.23	0.21	0.20	0.15	0.15	0.02	**0.004**	0.261	0.039	0.131
	Anaerovibrio	0.12	0.09	0.11	0.09	0.09	0.16	0.01	0.799	0.391	0.963	**0.071**
	RFN20	0.18	0.24	0.26	0.34	0.30	0.31	0.03	**0.017**	0.002	0.129	0.670
**Proteobacteria**	Succinivibrio	0.10	0.06	0.10	0.05	0.04	0.07	0.01	**0.074**	0.123	0.581	0.590
**Lentisphaerae**	Victivallaceae family	0.72	1.07	1.14	1.30	1.37	1.09	0.20	0.406	**0.078**	0.444	0.904
**Chloroflexi**	Family Anaerolinaceae; Genus SHD-231	0.13	0.20	0.21	0.27	0.29	0.23	0.03	**0.072**	0.119	0.208	0.882

^1^ Data is shown as LS Means with standard errors

^2^ n = 4 among groups.

Bold P-values indicate genera or family that tend to differ (P < 0.1) and significantly differ (P < 0.05) between feeding systems

### Correlations between ruminal methane production, metabolites and bacterial abundance

**Fig 3:**
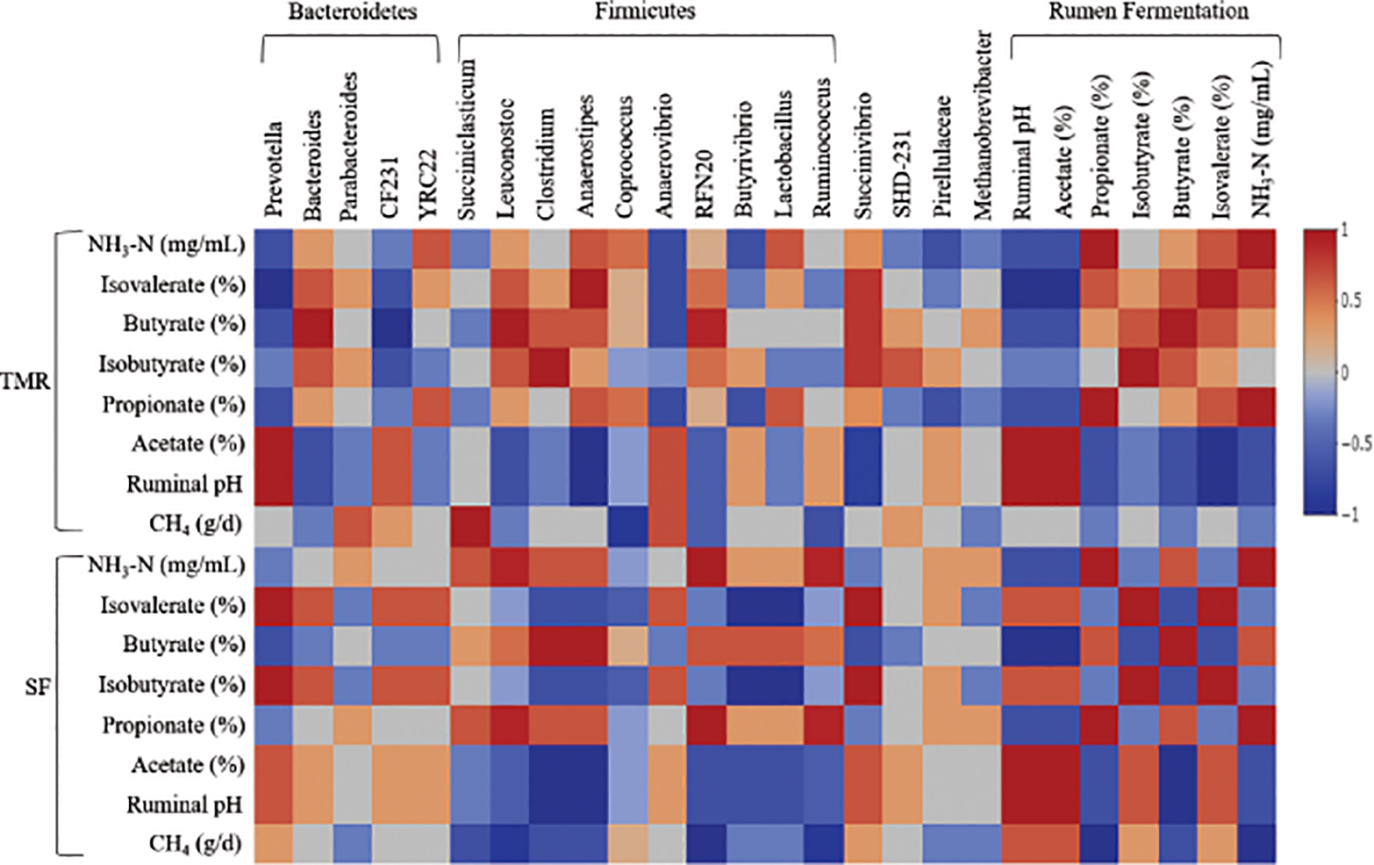
Correlation between efficiency parameter and genus abundance. Kendall’s non parametric correlation matrix of the dominant bacterial genera across the rumen samples. The genera were included in the matrix if they were in at least 50% of the steers and represented at least 0.1% of the bacterial community in at least one of the steers. Mean values of different time intervals were included for both microbial abundance and production parameters. Strong correlations are indicated by the intensity of the color. The scale colors denote whether the correlation is positive (closer to 1, red squares) or negative (closer to -1, blue squares) between the genera and the efficiency parameters.

*Prevotella*, the most abundant genus in both the feeding system (up to 26%), showed a negative (Kendall’s τ =  −1, *P* < 0.001) and positive (Kendall’s τ = 1, *P* < 0.001) correlation with isofatty acids in TMR and SF respectively (S4–S7 Tables; Fig 3). It also exerted a positive (Kendall’s τ = 1, *P* < 0.001) correlation with ruminal pH and acetate in TMR feeding system. The next most abundant genera *Ruminococcus* and *Butyrivibrio* did not exert any correlation with any of the production variables in both the feeding system. However, *Ruminococcus* tended to exert a negative (Kendall’s τ = −0.91, *P* = 0.087) and positive (Kendall’s τ = 0.91, *P* = 0.087) correlation with CH_4_ production and propionate proportion respectively in SF system. The differentially expressed genera *RFN20* and *Succiniclasticum* had a strong negative (Kendall’s τ = −1, *P* < 0.001) and positive (Kendall’s τ = 1, *P* < 0.001) correlation with CH_4_ production in SF and TMR respectively. Considering the rumen metabolites, propionate and NH_3_-N concentration in SF system correlated negatively (Kendall’s τ = -1, *P* < 0.001) with the methane production.

## Discussion

### Effects of feeding system on CH_4_ emissions

In our experiment, feeding TMR resulted in increases in CH_4_ production (absolute) and CH_4_ yield, although the intake amount was same as for SF. The observed DMI to meet nutrient requirements for an average 0.65 kg daily gain were slightly higher than those predicted by the Korean feeding standard for beef cattle [25]. But, the CH_4_ yield (g/kg DMI) in the current experiment was observed to be much lower than those reported earlier [26] [27]. However, this can be explained by the high feed quality and proportion of concentrate level in the feed in Korea. Similar CH_4_ yield (g/kg DMI) was noted in an earlier report from Korea [28]. A strong relationship between DMI and ruminal CH_4_ production has been reported [29] [30]. This indicates that increasing the DMI resulted in increased fermentable substrate, including both structural and nonstructural carbohydrates [31]. However, there is evidence that increasing the feed intake decreased the CH_4_ yield [32] [33] [34], which was explained by the decrease in the mean rumen retention time, which consequently decreased the extent of rumen fermentation compared to low intake levels [35] [36] [37]. This is why our experiment was performed at a restricted feed intake level and not ad libitum using TMR and SF.

According to a survey by Heinrichs et al. [38], only 7.1% of the particles in TMR were greater than 19 mm versus 16–18% for various forages, which is consistent with our results of 54 and 181 g/kg DM that is 5.4% and 18.1% for TMR and SF, respectively. Liu et al. [39] reported that TMR feeding had a greater proportion of ruminal contents with particle size < 1.18 mm, which is the critical size for particles to pass the rumen [40]. Although reducing forage particle size during the TMR mixing process usually results in reduced rumen solid retention time, the effects on DMI and digestibility remain less clear. Kononoff and Heinrichs [41] found that DM digestibility decreased with increasing ration particle size, whereas Kononoff and Heinrichs [42] reported the opposite. There were no differences (P > 0.05) in the indirect total digestibility of DM, OM, CP, and aNDFom and intake energy between the feeding systems in our experiment, and numerous previous studies have also found no significant differences in DMI and nutrient digestibility between the two feeding methods [8] [10] [41]. These different results are likely the result of interactions between forage particle sizes and forage type and the forage-to-concentrate ratio [40]. The limited effects of passage rate on total tract digestibility could also have been due to postruminal compensatory digestion [43]. Reducing the particle size distribution with feed processing might be another strategy for decreasing CH_4_ emissions because it probably alters the rate of fermentation and passage rate of the particles. Recently, Huhtanen et al. [37] observed an inverse relationship between the rate of feed passage and CH_4_ production. This relationship was also seen by Okine et al. [44], who found that ruminal passage rate constants and ruminal fluid dilution rates explain 28% and 25% of the variation in methane, respectively. Although we did not measure the passage rate of the feed, perhaps the increased methane production is not associated with the decreased particle size of TMR. However, the diurnal variation in methane production between the feeding systems shows a higher production of CH_4_ in TMR system after feeding (S1 Fig).

### Effects of feeding systems on rumen fermentation characteristics and bacterial abundance

In this study, our results suggested that the TMR or SF system did not influence the abundance of the major microbiome in the rumen of Holstein steers, which may be a result of feeding the same diet ingredients. However, a change in rumen fermentation pattern between the feeding systems was observed, leading to an increase in ruminal pH after feeding TMR. In general, a reduced forage particle size decreases the time spent chewing and creates a trend toward decreased ruminal pH due to increased availability of substrate for fermentation [45]. However, this was not the case with TMR feeding in our experiment. It is unlikely that the reduced particle size due to the TMR mixing process was the factor determining the reduction in ruminal pH and CH_4_ production. Feeding TMR eliminates the need to feed large meals of concentrate, which may be beneficial in terms of maintaining a high ruminal pH [5], consistent with our findings. In our study, steers fed SF showed a consistent decrease in pH from 6.7 to 6.3 until 4.5 h post feeding, which might be attributed to the rapid consumption of concentrate that was fed 40 min after roughage was fed. It is generally accepted that ruminal CH_4_ production is lower when the ruminal pH is low, as in high-grain diets that are rich in soluble carbohydrate or starch rather than those that include a high amount of forage [46]. The same phenomena was observed in recent reports where the animals were fed high grain diets [47] [48]. Nevertheless, Hünerberg et al. [49] reported a discrepant result that reductions in diurnal ruminal pH did not correlate with the reduction in CH_4_ production when ruminal pH decreased to threshold levels for subacute (5.2 ≤ pH < 5.5) or acute (pH < 5.2) ruminal acidosis. This is also in agreement with the observed no correlation between ruminal pH and CH_4_ production in the current experiment.

The overall ruminal fermentation characteristics in this experiment indicated that the fermentation pattern of steers fed TMR shifted away from propionate towards acetate. Moss et al. [50] reported that the production of acetate and butyrate from pyruvate is accompanied by the production of H_2_, whereas propionate production utilise H_2_, which is the major substrate for methanogenesis. The increase in CH_4_ with the TMR system in our experiment might be due to an increase in acetate from cellulose digestion, although no difference in total NDF digestion was observed in this experiment, and a shift in the metabolic H_2_ sink toward the production of acetate after feeding, which consequently increased the acetate: propionate ratio (A/P). This is also supported by Li et al. [51], who observed an increase in activity of xylanase, the most active fibrolytic enzyme, in the TMR feeding system compared to SF. This could be the major reason for the observed increase in CH_4_ in TMR system in the current experiment. However, the butyrate proportion in the rumen is similar between the feeding systems after 1.5 h of feeding, though the abundance of *Butyrivibrio* in TMR was higher than SF feeding system, which had been reported to involve in decomposition of hemicellulose and cellulose thereby producing huge amount of butyrate [52], majorly contributing to ruminal CH_4_ production [50]. The pattern of ruminal fermentation for TMR feeding in this experiment contrasts with other reports: decrease in A/P [51] and no difference in VFA and A/P [8] [39] compared to the SF system.

*Bacteroidetes* and *Firmicutes* were the most abundant phylum in the present study irrespective of the feeding system, and the results were found to be similar to that of several other studies fed high concentrate diets [53] [54] [55]. *Prevotella*, the most abundant genera in the phylum *Bacteroidetes*, was not found to vary between the feeding systems in the current experiment. However, observed strong positive and negative correlations of *Prevotella* with acetate and propionate respectively, in TMR, might give another explanation for the increased production of acetate. *Prevotella* was also widely noted in animals fed high concentrate diets [49], which comprise a well-known xylan degrading group [56]. Furthermore, some of the species in *Prevotella* are also efficient hemicellulose, cellulose, pectin, long-chain carbohydrate, and protein digesters [57] [58] [59], which implies their important role in digestion. It has been suggested that, this bacterial family contributes to fumarate reductase activity, which could produce propionate via succinate or acrylate pathway [60], but the effect of *Prevotella* on the rumen fermentation and their relationship with methane production have not yet been clarified, because uncultured *Prevotella* represent a large portion of the bacterial population. It is worth noting that animals fed by SF system, that emitted lower CH_4,_ also showed a strong negative correlation between *Prevotella* and the proportion of propionate in this experiment.

Higher abundance of *RFN20 (Erysipelotrichaceae)* in TMR system that positively correlated with the CH_4_ production in earlier studies [61], contrastingly exhibited no correlation in the current experiment. On the other hand, *RFN20* had a significantly strong negative and positive correlation with CH_4_ and propionate proportion, respectively, in SF system. It was further coincided with the significantly higher abundance of *Coprococcus* of phylum *Firmicutes* for SF system. *Coprococcus* was also independently found to be enriched in the efficient animals' microbiome [62], which use H_2_ for the production of propionate through the acrylate pathway that utilizes lactate [63]. However, no strong correlation of *Coprococcus* with propionate or CH_4_ production was observed in SF system. In addition, low CH_4_ production in SF system were also supported by the strong negative correlation with the abundance of *Succiniclasticum*, which is specialized in fermenting succinate and converting it to propionate as a major fermentation product [64] [65].

Ammonia-N is produced by the deamination and fermentation of the peptides released during protein digestion [66], which leads to a higher ratio of iso-fatty acids [67]. There is much interest in the importance of iso-fatty acids in the rumen, because isobutyric, isovaleric, and 2-methylbutyric acids are required for resynthesis of the branched-chain amino acids by carboxylation and amination [67] [68] [69]. The maximum ruminal NH_3_-N concentration in our experiment occurred at 3 h after feeding in steers fed SF. This implies that the supply of available nitrogen in the rumen is relatively well synchronised with the slow release of energy for microbial protein synthesis for SF compared to TMR. There is little experimental evidence to support the synchrony of energy and nitrogen release in the rumen, although Kim et al. [70] demonstrated that altering the degree of synchrony in the rates of ruminal release of energy and nitrogen had a marked effect on microbial protein synthesis when the diet contained about 30% DM as fermentable carbohydrate. Although microbial protein synthesis in the rumen was not determined in our experiment, the 3 or 1.3 times higher concentrations of isobutyrate and isovalerate in SF might have led to greater efficiency of microbial protein synthesis in the later phases of feeding. This is further supported by Kim et al. [71], who demonstrated that iso-fatty acids had a positive correlation with the efficiency of microbial growth. Hungate [72] also found that the incorporation of peptides synthesised from iso-fatty acids into microbial cells resulted in a net consumption of H_2_. Beever [73] suggested that the dry matter partitioning between microbial protein synthesis and fermentation influences hydrogen production and hence methanogenesis. In accordance with it, a strong negative relationship was observed between NH_3_-N and CH_4_ in SF system.

The relationship between numbers of methanogens and amount of CH_4_ produced has been a topic of debate. However, in our study, population structure of the methanogens could not explain the difference in CH_4_ production between the feeding systems. These results agree with previous studies that also showed that population of methanogens were not significantly different between two groups of feedlot bulls [74] and two groups of lambs [75] that produced significantly different amounts of CH_4_. Furthermore, the abundance of major genera belonging to *Bacteroidetes*, *Firmicutes*, and *Euryarchaeota* that are mainly involved in methane production did not exert any differences between the feeding systems. The shifts noted in other minor groups such as *Coprococcus*, *Succiniclasticum*, *Butyrivibrio*, and *Succinivibrio* provide novel insights, since their abundance were associated with the changes in ruminal VFA synthesis or methane production. However, the reason for the variation of abundance of these minor genera cannot be explained beyond the change in ruminal fermentation pattern as witnessed upon time after feeding. Despite no direct effect on methanogens and other major microbiome, the variation in abundance of minor microbiome that developed with the two feeding system probably varied in their metabolic potential, resulting in different proportion of metabolites becoming available for downstream methanogenic activity thereby altering the CH_4_ production. The unclassified *Clostridiales*, *Bacteroidales* and *Ruminococcaceae* alone corresponded to almost 30% of total population which were observed to be the core microbiome in rumen across the world [76], and these unclassified orders were reported to play an important role in biohydrogenation [77]. This implies that there are a lot of microbe in rumen that are needed to be characterized to open novel insights to further understand methanogenesis.

## Conclusions

This study demonstrated that the conventional method of feeding roughage and concentrates separately, reduces CH_4_ production without altering the efficiency of nutrient utilisation and major rumen microbiome. There was no evidence to support concerns that the difference in methane production between TMR and SF differed due to different underlying major rumen microbial population, but a cardinal point that emerges from our findings is that the functional characteristics of minor microbiota can have a large impact on ecosystem functioning, and fermentation pattern in the rumen.

## Supporting information

S1 Fig Diurnal methane production pattern of steers fed roughage and concentrate either as total mixed ration (dashed line; -●-) or separately (solid line; ■) measured after feeding. Error bar indicates standard error.
(TIF)

S1 Table **Average body weight and nutrient intake (A) and coefficient of digestibility of nutrients (B) of the steers fed roughage and concentrate either as total mixed ration (TMR) or separately (SF).** The values are expressed as least square means with standard error and n = 6.
(DOCX)

S2 Table Effect of feeding system on alpha diversity indices. Values are mean with standard deviation (SD).
(DOCX)

S3 Table Relative abundance of taxa in the two groups representing > 0.1% of total sequences. Data is shown as LS Means with standard errors with n = 4 among groups. Bold P-values indicate groups that tend to differ (P < 0.1) and significantly differ (P < 0.05).
(DOCX)

S4 Table Correlation coefficients between efficiency parameter and genus abundance in steers fed SF. Kendall’s non parametric correlation matrix of the dominant bacterial genera across the rumen samples. The genera were included in the matrix if they were in at least 50% of the steers and represented at least 0.1% of the bacterial community in at least one of the steers.
(XLS)

S5 Table P values of correlation coefficients between efficiency parameter and genus abundance in steers fed SF. Bold P-values indicate groups that tend to differ (P < 0.1) and significantly differ (P < 0.05).
(XLSX)

S6 Table Correlation coefficients between efficiency parameter and genus abundance in steers fed TMR. Kendall’s non parametric correlation matrix of the dominant bacterial genera across the rumen samples. The genera were included in the matrix if they were in at least 50% of the steers and represented at least 0.1% of the bacterial community in at least one of the steers.
(XLSX)

S7 Table P values of correlation coefficients between efficiency parameter and genus abundance in steers fed TMR. Bold P-values indicate groups that tend to differ (P < 0.1) and significantly differ (P < 0.05).
(XLSX)
