# Supplementary material for: Ruminal methane emissions, metabolic, and microbial profile of Holstein steers fed forage and concentrate, separately or as a total mixed ration
Source: PLoS One. 2018 Aug 15;13(8):e0202446. doi: 10.1371/journal.pone.0202446 (PMC6093700; doi:10.1371/journal.pone.0202446)
Supplement: S1 Table — Average body weight and nutrient intake (A) and coefficient of digestibility of nutrients (B) of the steers fed roughage and concentrate either as total mixed ration (TMR) or separately (SF). The values are expressed as least square means with standard error and n = 6. (DOCX) [file pone.0202446.s002.docx]

**S1 Table. (A) Average body weight and nutrient intake of the steers fed roughage and concentrate either as total mixed ration (TMR) or separately (SF) (*n* = 6)**

| Mean BW (kg) | | ADG (kg) | Nutrient intake (kg/d) | | | GEI (MJ/d) |
| --- | --- | --- | --- | --- | --- | --- |
| Initial | Final |  | DM | CP | NDF |  |
| 540 (± 34) | 572 (± 37) | 0.65 (± 0.11) | 8.43 (± 1.45) | 1.4 (± 0.24) | 2.42 (± 0.42) | 137 (± 23.6) |

Values in the bracket indicates standard deviation

**S1 Table. (B) Coefficient of digestibility of nutrients in the steers fed roughage and concentrate either as total mixed ration (TMR) or separately (SF) (*n* = 6)**

|  | TMR | SF | SEM | *P*-value |
| --- | --- | --- | --- | --- |
| DM | 0.60 | 0.60 | 0.15 | 0.809 |
| OM | 0.62 | 0.62 | 0.04 | 0.107 |
| CP | 0.64 | 0.65 | 0.15 | 0.369 |
| NDF | 0.35 | 0.34 | 0.02 | 0.560 |
| Energy, MJ/d | 78.09 | 79.46 | 2.00 | 0.720 |

ADG, average daily gain; GEI, gross energy intake; DM, dry matter; OM, organic matter;

CP, crude protein; NDF, neutral detergent fibre
