# Supplementary material for: Ruminal methane emissions, metabolic, and microbial profile of Holstein steers fed forage and concentrate, separately or as a total mixed ration
Source: PLoS One. 2018 Aug 15;13(8):e0202446. doi: 10.1371/journal.pone.0202446 (PMC6093700; doi:10.1371/journal.pone.0202446)
Supplement: S2 Table — Values are mean with standard deviation (SD). (DOCX) [file pone.0202446.s003.docx]

| **Indices** | **SF** | | | | | | **TMR** | | | | | | **P value**  **(Feed)** | **P value (Time)** | | |
| --- | --- | --- | --- | --- | --- | --- | --- | --- | --- | --- | --- | --- | --- | --- | --- | --- |
|  | **1.5 h** | | **3 h** | | **4.5 h** | | **1.5 h** | | **3 h** | | **4.5 h** | |  | **1.5 h** | **3 h** | **4.5 h** |
|  | **Mean** | **SD** | **Mean** | **SD** | **Mean** | **SD** | **Mean** | **SD** | **Mean** | **SD** | **Mean** | **SD** |  |  |  |  |
| Dominance_D | 0.11 | 0.06 | 0.09 | 0.04 | 0.10 | 0.07 | 0.09 | 0.03 | 0.10 | 0.03 | 0.12 | 0.06 | 0.845 | 0.444 | 0.810 | 0.405 |
| **Simpson_1-D** | 0.89 | 0.06 | 0.91 | 0.04 | 0.90 | 0.07 | 0.91 | 0.03 | 0.90 | 0.03 | 0.88 | 0.06 | 0.843 | 0.444 | 0.809 | 0.406 |
| **Shannon_H** | 3.12 | 0.31 | 3.14 | 0.20 | 3.14 | 0.36 | 3.18 | 0.19 | 3.06 | 0.23 | 3.07 | 0.37 | 0.500 | 0.705 | 0.541 | 0.409 |
| **Evenness_e^H/S** | 0.09 | 0.03 | 0.10 | 0.02 | 0.10 | 0.02 | 0.11 | 0.02 | 0.09 | 0.02 | 0.09 | 0.03 | 0.747 | 0.462 | 0.290 | 0.397 |
| Brillouin | 1.39 | 0.20 | 1.44 | 0.11 | 1.39 | 0.27 | 1.48 | 0.18 | 1.45 | 0.14 | 1.31 | 0.15 | 0.475 | 0.550 | 0.919 | 0.303 |
| Menhinick | 25.00 | 2.75 | 22.40 | 2.39 | 24.40 | 2.28 | 22.80 | 2.19 | 25.28 | 4.80 | 24.13 | 3.52 | **0.022** | **0.034** | 0.129 | 0.864 |
| Margalef | 57.77 | 6.31 | 51.71 | 5.78 | 56.50 | 5.76 | 52.58 | 5.10 | 58.11 | 11.14 | 56.01 | 8.88 | **0.019** | **0.031** | 0.127 | 0.894 |
| Equitability_J | 0.57 | 0.05 | 0.58 | 0.03 | 0.57 | 0.06 | 0.59 | 0.04 | 0.55 | 0.04 | 0.56 | 0.06 | 0.934 | 0.517 | 0.350 | 0.317 |
| Berger-Parker | 0.25 | 0.12 | 0.22 | 0.09 | 0.23 | 0.13 | 0.19 | 0.07 | 0.23 | 0.05 | 0.26 | 0.13 | 0.785 | 0.431 | 0.770 | 0.400 |
| **Chao-1** | 250.00 | 27.48 | 224.00 | 23.93 | 244.00 | 22.82 | 228.00 | 21.91 | 252.75 | 47.97 | 241.25 | 35.21 | **0.024** | **0.034** | 0.129 | 0.864 |

**S2 Table. Effect of feeding system on alpha diversity indices.**
