# Supplementary material for: Ruminal methane emissions, metabolic, and microbial profile of Holstein steers fed forage and concentrate, separately or as a total mixed ration
Source: PLoS One. 2018 Aug 15;13(8):e0202446. doi: 10.1371/journal.pone.0202446 (PMC6093700; doi:10.1371/journal.pone.0202446)
Supplement: S3 Table — Data is shown as LS Means with standard errors with n = 4 among groups. Bold P-values indicate groups that tend to differ (P < 0.1) and significantly differ (P < 0.05). (DOCX) [file pone.0202446.s004.docx]

**S3 Table. Relative abundance of taxa in the two groups representing > 0.1% of total sequences**

| **Phylum** | **Classification** | **Percentage of total sequences ^1,2^** | | | | | | **SEM** | **P value (Feed )** | **P value (Time)** | | |
| --- | --- | --- | --- | --- | --- | --- | --- | --- | --- | --- | --- | --- |
|  |  | **SF** | | | **TMR** | | |  |  |  |  |  |
|  |  | **1.5 h** | **3 h** | **4.5 h** | **1.5 h** | **3 h** | **4.5 h** |  |  | **1.5 h** | **3 h** | **4.5 h** |
| **Bacteroidetes** |  | 44.97 | 44.46 | 43.73 | 39.36 | 44.40 | 49.12 | 3.86 | 0.985 | 0.686 | 0.990 | 0.543 |
|  | Prevotella | 22.99 | 19.46 | 18.03 | 16.26 | 20.12 | 26.84 | 2.53 | 0.861 | 0.622 | 0.928 | 0.441 |
|  | Bacteroides | 1.35 | 1.05 | 1.44 | 1.04 | 0.88 | 1.58 | 0.13 | 0.687 | 0.658 | 0.469 | 0.835 |
|  | Parabacteroides | 0.22 | 0.30 | 0.21 | 0.17 | 0.15 | 0.16 | 0.02 | **0.081** | 0.336 | **0.026** | **0.084** |
|  | Paludibacter | 0.12 | 0.13 | 0.20 | 0.15 | 0.14 | 0.19 | 0.02 | 0.723 | 0.573 | 0.807 | 0.943 |
|  | Family Paraprevotellaceae; Genus CF231 | 0.88 | 0.88 | 0.82 | 0.71 | 0.58 | 0.67 | 0.06 | **0.026** | 0.549 | 0.214 | 0.266 |
|  | Family Paraprevotellaceae; Genus YRC22 | 0.90 | 0.50 | 0.29 | 0.26 | 0.29 | 0.55 | 0.07 | **0.082** | 0.155 | 0.112 | 0.184 |
|  | Family Bacteroidaceae;  Genus 5-7N15 | 0.30 | 0.32 | 0.34 | 0.31 | 0.24 | 0.36 | 0.01 | 0.600 | 0.939 | 0.132 | 0.780 |
|  | Family Rikenellaceae | 0.14 | 0.16 | 0.18 | 0.15 | 0.11 | 0.17 | 0.01 | 0.404 | 0.846 | 0.276 | 0.834 |
|  | Order Bacteroidales;  Family BS11 | 4.06 | 2.78 | 4.11 | 4.20 | 4.71 | 3.11 | 0.39 | 0.615 | 0.912 | 0.124 | 0.599 |
|  | Order Bacteroidales;  Family S24-7 | 2.64 | 3.51 | 2.87 | 1.90 | 2.02 | 1.85 | 0.56 | 0.138 | 0.350 | 0.457 | 0.550 |
|  | Order Bacteroidales;  Family RF16 | 0.90 | 0.98 | 0.73 | 0.69 | 0.86 | 1.05 | 0.11 | 0.967 | 0.621 | 0.805 | 0.335 |
|  | Bacteroidales Unclassified | 9.56 | 13.58 | 13.76 | 12.74 | 13.72 | 11.75 | 0.91 | 0.777 | 0.211 | 0.961 | 0.635 |
| **Firmicutes** |  | 37.60 | 36.52 | 36.79 | 42.90 | 38.05 | 36.36 | 1.61 | 0.466 | 0.526 | 0.363 | 0.935 |
|  | Ruminococcus | 2.53 | 3.33 | 3.94 | 3.32 | 3.35 | 2.39 | 0.31 | 0.613 | 0.463 | 0.987 | 0.233 |
|  | Butyrivibrio | 1.74 | 1.29 | 1.15 | 3.31 | 3.38 | 2.21 | 0.67 | **0.047** | 0.409 | 0.179 | 0.425 |
|  | Lactobacillus | 1.99 | 2.01 | 1.73 | 2.19 | 0.97 | 2.59 | 0.16 | 0.982 | 0.625 | 0.119 | 0.441 |
|  | Oscillospira | 0.64 | 0.55 | 0.67 | 0.68 | 0.51 | 0.63 | 0.09 | 0.902 | 0.857 | 0.838 | 0.861 |
|  | Succiniclasticum | 0.95 | 0.84 | 0.34 | 0.23 | 0.41 | 0.54 | 0.11 | **0.063** | 0.132 | 0.514 | 0.139 |
|  | Streptococcus | 0.55 | 0.91 | 0.38 | 1.03 | 0.69 | 0.71 | 0.09 | 0.285 | 0.229 | 0.547 | 0.103 |
|  | Leuconostoc | 0.38 | 0.38 | 0.35 | 0.40 | 0.28 | 0.57 | 0.02 | 0.457 | 0.571 | 0.328 | 0.181 |
|  | Weissella | 0.27 | 0.28 | 0.25 | 0.28 | 0.19 | 0.37 | 0.02 | 0.800 | 0.778 | 0.289 | 0.259 |
|  | Mogibacterium | 0.14 | 0.11 | 0.16 | 0.16 | 0.17 | 0.14 | 0.02 | 0.640 | 0.870 | 0.342 | 0.804 |
|  | Clostridium | 0.36 | 0.41 | 0.45 | 0.45 | 0.34 | 0.32 | 0.03 | 0.458 | 0.186 | 0.364 | 0.155 |
|  | Anaerostipes | 0.34 | 0.44 | 0.65 | 0.42 | 0.38 | 0.25 | 0.08 | 0.246 | 0.753 | 0.760 | 0.112 |
|  | Blautia | 0.22 | 0.14 | 0.21 | 0.23 | 0.17 | 0.18 | 0.01 | 0.967 | 0.837 | 0.117 | 0.345 |
|  | Coprococcus | 0.28 | 0.23 | 0.21 | 0.20 | 0.15 | 0.15 | 0.02 | **0.004** | 0.261 | 0.039 | 0.131 |
|  | Dorea | 0.13 | 0.13 | 0.15 | 0.14 | 0.10 | 0.14 | 0.01 | 0.718 | 0.740 | 0.434 | 0.932 |
|  | Faecalibacterium | 0.17 | 0.16 | 0.21 | 0.13 | 0.10 | 0.21 | 0.02 | 0.437 | 0.289 | 0.327 | 0.940 |
|  | Anaerovibrio | 0.12 | 0.09 | 0.11 | 0.09 | 0.09 | 0.16 | 0.01 | 0.799 | 0.391 | 0.963 | **0.071** |
|  | Phascolarctobacterium | 0.13 | 0.12 | 0.15 | 0.12 | 0.11 | 0.14 | 0.01 | 0.657 | 0.839 | 0.695 | 0.944 |
|  | Family Clostridiaceae;  Genus SMB53 | 0.07 | 0.18 | 0.31 | 0.20 | 0.20 | 0.27 | 0.05 | 0.731 | 0.025 | 0.916 | 0.794 |
|  | Family Erysipelotrichaceae;  Genus p-75-a5 | 0.25 | 0.24 | 0.25 | 0.26 | 0.28 | 0.24 | 0.03 | 0.732 | 0.957 | 0.576 | 0.957 |
|  | Family Erysipelotrichaceae;  Genus RFN20 | 0.18 | 0.24 | 0.26 | 0.34 | 0.30 | 0.31 | 0.03 | **0.017** | 0.002 | 0.129 | 0.670 |
|  | Family Mogibacteriaceae | 0.67 | 0.63 | 0.67 | 0.65 | 0.63 | 0.48 | 0.04 | 0.393 | 0.925 | 0.973 | 0.116 |
|  | Family Christensenellaceae | 1.27 | 0.93 | 0.86 | 1.63 | 1.28 | 0.87 | 0.17 | 0.392 | 0.660 | 0.145 | 0.987 |
|  | Family Clostridiaceae | 0.28 | 0.33 | 0.31 | 0.36 | 0.27 | 0.33 | 0.02 | 0.722 | 0.022 | 0.281 | 0.837 |
|  | Family Lachnospiraceae | 3.35 | 3.38 | 3.39 | 4.16 | 3.17 | 3.54 | 0.34 | 0.616 | 0.620 | 0.612 | 0.764 |
|  | Family Peptostreptococaceae | 0.10 | 0.21 | 0.21 | 0.25 | 0.14 | 0.21 | 0.03 | 0.594 | 0.069 | 0.192 | 0.975 |
|  | Family Ruminococcaceae | 9.03 | 7.58 | 8.40 | 9.38 | 7.98 | 7.56 | 0.36 | 0.968 | 0.867 | 0.592 | 0.267 |
|  | Family Veillonellaceae | 0.54 | 0.65 | 0.55 | 0.44 | 0.43 | 0.46 | 0.06 | 0.112 | 0.523 | 0.272 | 0.600 |
|  | Clostridiales unclassified | 9.27 | 9.41 | 9.07 | 10.45 | 10.72 | 7.95 | 0.49 | 0.631 | 0.548 | 0.410 | 0.521 |
| **Verrucomicrobia** |  | 4.04 | 6.85 | 5.83 | 5.38 | 5.13 | 4.23 | 0.94 | 0.596 | 0.660 | 0.547 | 0.481 |
|  | Akkermansia | 0.21 | 0.12 | 0.26 | 0.16 | 0.10 | 0.20 | 0.02 | 0.360 | 0.639 | 0.391 | 0.640 |
|  | Order LD1-PB3 | 0.03 | 0.04 | 0.03 | 0.24 | 0.34 | 0.13 | 0.10 | 0.134 | 0.430 | 0.431 | 0.405 |
|  | Family RFP12 | 3.49 | 6.45 | 5.31 | 4.82 | 4.54 | 3.75 | 0.86 | 0.533 | 0.640 | 0.455 | 0.498 |
| **Actinobacteria** |  | 3.06 | 2.29 | 2.85 | 1.97 | 2.21 | 1.21 | 0.47 | 0.157 | 0.538 | 0.937 | 0.061 |
|  | Family Bifidobacteriaceae | 0.22 | 0.33 | 0.37 | 0.13 | 0.22 | 0.40 | 0.07 | 0.727 | 0.725 | 0.727 | 0.954 |
|  | Family Coriobacteriaceae | 2.58 | 1.80 | 2.17 | 1.68 | 1.80 | 0.65 | 0.46 | 0.213 | 0.604 | 0.989 | 0.204 |
| **Tenericutes** |  | 2.65 | 2.03 | 2.40 | 2.21 | 2.02 | 1.80 | 0.25 | 0.379 | 0.725 | 0.988 | 0.296 |
|  | Anaeroplasma | 0.10 | 0.12 | 0.08 | 0.14 | 0.09 | 0.09 | 0.01 | 0.787 | 0.444 | 0.569 | 0.793 |
|  | Class Mollicutes;  Order RF39 | 2.47 | 1.80 | 2.20 | 1.99 | 1.81 | 1.63 | 0.24 | 0.380 | 0.710 | 0.991 | 0.302 |
| **Proteobacteria** |  | 2.45 | 1.69 | 1.94 | 1.89 | 1.59 | 1.96 | 0.20 | 0.494 | 0.395 | 0.767 | 0.981 |
|  | Desulfovibrio | 0.75 | 0.61 | 0.56 | 0.54 | 0.50 | 0.56 | 0.10 | 0.458 | 0.634 | 0.576 | 0.994 |
|  | Succinivibrio | 0.10 | 0.06 | 0.10 | 0.05 | 0.04 | 0.07 | 0.01 | **0.074** | 0.123 | 0.581 | 0.590 |
|  | Family Enterobacteriaceae | 0.48 | 0.32 | 0.37 | 0.48 | 0.32 | 0.49 | 0.07 | 0.716 | 0.991 | 1.000 | 0.343 |
| **Cyanobacteria** |  | 1.08 | 1.72 | 1.59 | 1.90 | 1.76 | 1.28 | 0.19 | 0.483 | **0.014** | 0.827 | 0.525 |
|  | Order YS2 | 0.96 | 1.62 | 1.45 | 1.74 | 1.61 | 1.04 | 0.12 | 0.640 | **0.004** | 0.966 | 0.490 |
| **Lentisphaerae** | Victivallaceae family | 0.72 | 1.07 | 1.14 | 1.30 | 1.37 | 1.09 | 0.20 | 0.262 | **0.078** | 0.444 | 0.904 |
| **Spirochaetes** |  | 1.04 | 0.95 | 0.95 | 0.88 | 0.85 | 1.01 | 0.14 | 0.739 | 0.690 | 0.485 | 0.894 |
|  | Treponema | 1.00 | 0.92 | 0.92 | 0.84 | 0.80 | 0.96 | 0.14 | 0.677 | 0.670 | 0.415 | 0.922 |
| **TM7** | Family F16 | 0.39 | 0.45 | 0.58 | 0.49 | 0.40 | 0.37 | 0.07 | 0.562 | 0.722 | 0.808 | 0.328 |
| **Planctomycetes** | Family Pirellulaceae | 0.35 | 0.18 | 0.36 | 0.20 | 0.22 | 0.14 | 0.03 | 0.120 | 0.316 | 0.429 | 0.159 |
| **Chloroflexi** | Family Anaerolinaceae; Genus SHD-231 | 0.13 | 0.20 | 0.21 | 0.27 | 0.29 | 0.23 | 0.03 | **0.072** | 0.119 | 0.208 | 0.882 |
| **Fibrobacteres** | Fibrobacter | 0.13 | 0.11 | 0.11 | 0.13 | 0.09 | 0.14 | 0.01 | 0.962 | 0.913 | 0.632 | 0.278 |
|  |  |  |  |  |  |  |  |  |  |  |  |  |
|  | **Archaea** |  |  |  |  |  |  |  |  |  |  |  |
| **Euryarchaeota** | Methanobrevibacter | 1.02 | 1.15 | 1.19 | 0.85 | 1.29 | 0.79 | 0.30 | 0.452 | 0.253 | 0.916 | 0.952 |
|  |  |  |  |  |  |  |  |  |  |  |  |  |

^1^ Data is shown as LS Means with standard errors

^2^ n = 4 among groups.

Bold values indicate groups that tend to differ (P < 0.1) and significantly differ (P < 0.05)
